# Reference intervals of echocardiographic measurements in healthy adult dairy goats

**DOI:** 10.1371/journal.pone.0183293

**Published:** 2017-08-22

**Authors:** Olga Szaluś-Jordanow, Michał Czopowicz, Lucjan Witkowski, Marcin Mickiewicz, Tadeusz Frymus, Iwona Markowska-Daniel, Emilia Bagnicka, Jarosław Kaba

**Affiliations:** 1 Department of Small Animal Diseases with Clinic, Division of Infectious Diseases, Warsaw University of Life Sciences-SGGW, Warsaw, Poland; 2 Laboratory of Veterinary Epidemiology and Economics, Warsaw University of Life Sciences-SGGW, Warsaw, Poland; 3 Institute of Genetics and Animal Breeding, Polish Academy of Sciences, Jastrzębiec, Poland; University of Bari, ITALY

## Abstract

**Objectives:**

To determine references intervals for echocardiographic measurements in adult dairy goats.

**Animals:**

125 clinically healthy, adult dairy goats aged 2–9 years, belonging to two breeds–Polish Fawn Improved (PFI, n = 64, weight range from 46 to 73, median of 58.5kg) and Polish White Improved (PWI, n = 61, weight range from 48 to 80 kg, median of 67.9kg), closely related to French Alpine and Saanen, respectively.

**Methods:**

Non-invasive transthoracic echocardiography examination was performed in unsedated goats in a standing position. Two-dimensional, M-mode and pulsed wave Doppler measurements were obtained. A non-parametric method was applied for determination of reference intervals. Measurements for the two breeds were compared using an analysis of covariance to control for their body weight. Repeatability was assessed using a between-day coefficient of variation and a coefficient of repeatability.

**Results:**

Following reference intervals were determined: aortic diameter in diastole 2.2–3.3, left atrial diameter in systole 2.5–4.3cm cm, the ratio of the left atrial diameter to the aortic diameter 0.96–1.5, right ventricular internal diameter in diastole 0.4–1.7cm, left ventricular internal diameter in systole and diastole 1.8–3.2 and 3.2–5.6 cm, respectively, inter-ventricular septum thickness in systole and diastole 0.7–1.5 and 0.5–1.1cm, respectively, left ventricular posterior-wall in systole and diastole 0.8–1.6 and 0.5–1.2cm, respectively, E-point to septal separation 0.3–0.8cm, left ventricular fractional shortening 28–54%, left ventricular ejection fraction 55–86%, maximum Left and Right Ventricular Outflow Tract velocity 80–140 cm/s and 70–130 cm/s, respectively Left and Right Ventricular Outflow Tract pressure gradient 2.5–8.9mmHg and 1.9–6.5mmHg, respectively. Most of the differences between the two breeds could be attributed to different body weight.

**Conclusions:**

The study provides echocardiographic reference intervals determined on the highest sample of apparently healthy goats so far enrolled.

## Introduction

Since the use of echocardiography in a goat was first reported in 1992[1] six studies regarding this subject have been carried out–one on Swedish domestic goats[2], one on Philippines native goats[3], three on Saneen goats [4–6] and one on a mixed sample of Saneen and Golden Guernsey goats[7]. They all proved that echocardiography was as a safe and convenient diagnostic test in goats like in any other animal species. Echocardiography plays little role in medical practice of farm animals. Heart diseases are rarely diagnosed and diseased animals remain in a herd as long as they are able to maintain an expected level of productivity. Emergence of clinical signs of the heart disease results rather in culling then medication. However, goats are not rarely kept for companionship as well as they are used as experimental animals [8, 9] also in human cardiology studies [10, 11]. This compels more individual approach to this species.

The numbers of animals enrolled in so far published studies were low: 8 goats [2, 3], 10 goats [5], 12 goats [4], 22 goats [6] and 40 goats [7]. The four latter studies aimed to determine reference values either of two-dimensional and M-mode echocardiographic measurements [4, 6] or pulsed wave Doppler measurements [5] or both [7]. It is obvious that sufficient number of participants is essential to establish reliable reference intervals. A minimum of 120 is typically mentioned as the figure which allows the use of preferable distribution-free nonparametric approach [12, 13]. Even though fewer individuals can also suffice provided that more sophisticated statistical analyses are employed [14] no reference intervals should be calculated unless at least 20 observations are available [15]. As only two of the studies so far conducted met the aforementioned criteria [6, 7] we decided to perform the study which will allow to determine reference intervals of echocardiographic measurements on a large sample of healthy adult dairy goats.

## Materials and methods

The study was carried out between June and August 2013 and 2014, and was approved by the III Local Ethics Commission regarding Experiments in Animals at the Warsaw University of Life Sciences-SGGW (Approval number: 89/2012/2012).

### Animals

Female dairy goats from four dairy farms were enrolled in the study. Two farms were located in the Wielkopolska province (52.551464, 15.646461; 52.676207,16.876352) and two were located in Mazovia province (52.031759, 20.828227; 51.773386. 21.362042). All the herd owners gave permission to conduct the study in their farms.

They belonged to two breeds–Polish Fawn Improved (PFI) and Polish White Improved (PWI).

To be initially enrolled in the study goats had to be more than 2 and less than 10 year-old, currently in lactation and apparently healthy at the moment of examination (normal appetite and milk yield, sternal and lumbar body score between 2 and 4 when score of 0–1 signified emaciation and score of 5 signified obesity [16], and no signs of subclinical mastitis). A complete physical examination and electrocardiography (ECG) using AsCARD Mr. Silver electrocardiograph (Aspel S.A., Poland) were performed in each goat before the echocardiography. Goats must have had a sinus heart rhythm in ECG, a heart rate from 70 to 150 bpm, and no cardiac murmurs on auscultation.

All animals were weighed and their age was recorded.

In 20 goats randomly selected (simple random sampling), the entire echocardiographic examination was repeated twice at one-day interval by the same echocardiographer (OSJ) to determine repeatability of measurements.

### Echocardiographic examinations

The goats were examined in their own barns and they were held in standing position preferably by their owners to make them as relaxed as possible. No sedation was used.

Hair was clipped from small square areas on both sides of the thorax between the 4-6^th^ intercostal spaces and the skin was cleaned with surgical spirit and covered with ultrasound coupling gel (Aquasonic, Adamed, Poland). All echocardiographic examinations were performed by one examiner (OSJ) using Mindray M7 diagnostic ultrasound system with Phased Array probe (P4-2s). Simultaneously, ECG was recorded using Mindray a built-in ECG module and on its basis diastolic and systolic frames were chosen. Diastolic left and right ventricular dimensions were measured at the beginning of the QRS complex, systolic dimensions were measured at the peak downward point of septal motion.

**Fig 1:**
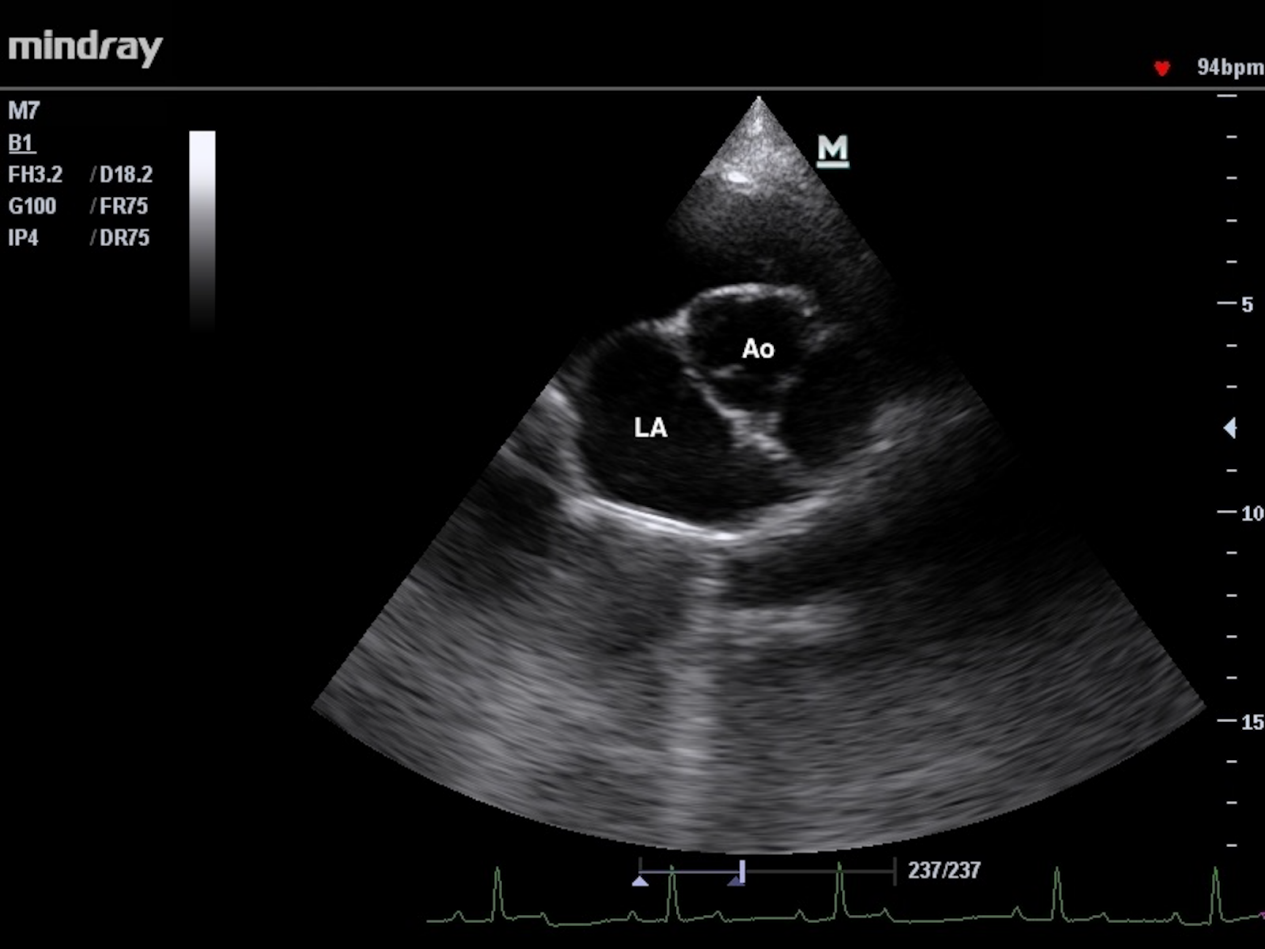
The right parasternal short-axis view of the heart base with aorta. LA- left atrium, Ao- aorta.

**Fig 2:**
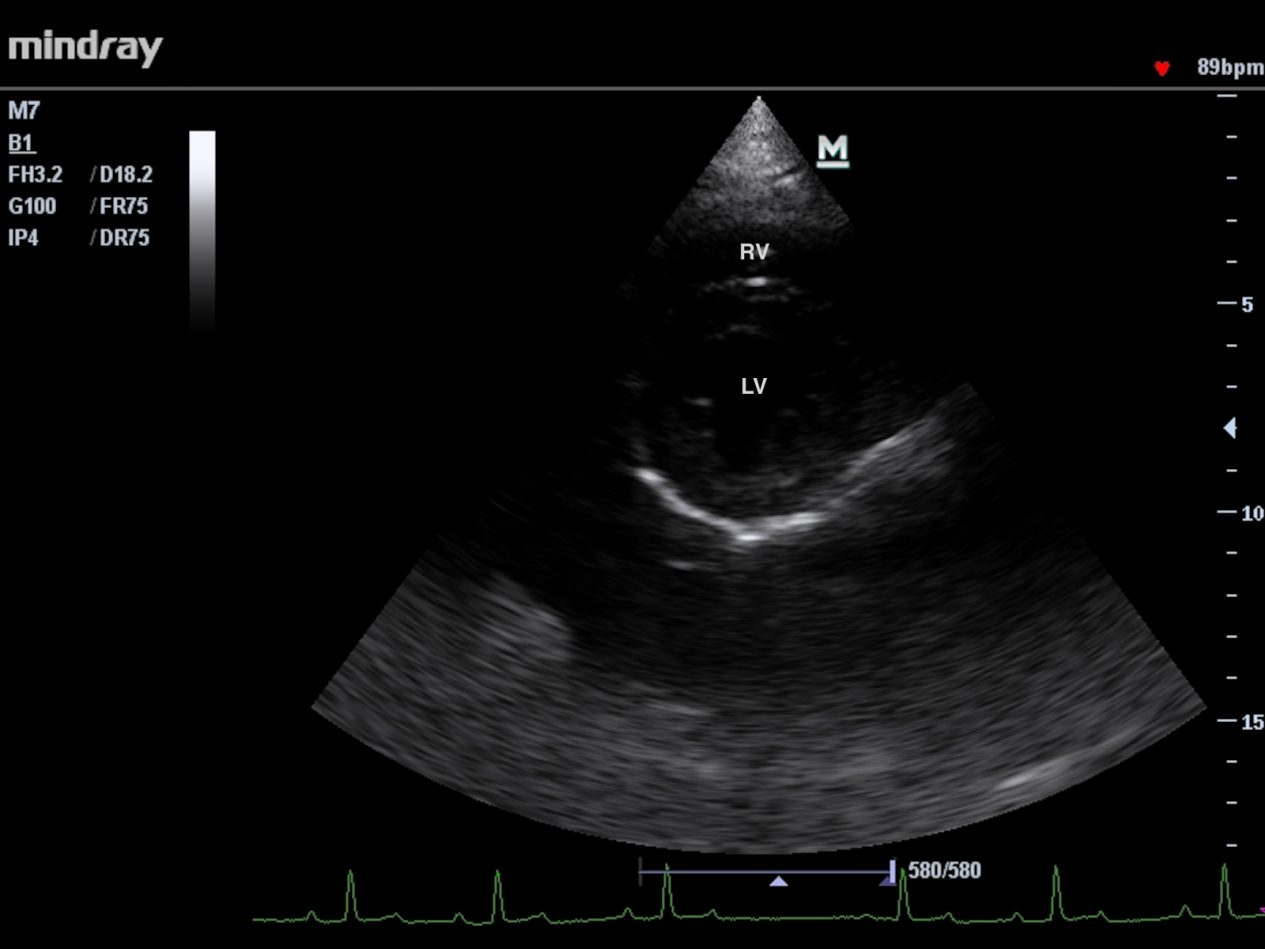
The right parasternal short-axis view of the left and right ventricles at the level of papillary muscles RV- right ventricle, LV- left ventricle.

Following views were obtained: right parasternal long axis view optimized for viewing the atria, right parasternal long axis view optimized for viewing the aorta, right parasternal short axis view optimized for: papillary muscle, chordae tendineae, mitral valve, aorta and main pulmonary artery, left parasternal 4 and 5 chambers view (Fig 1, Fig 2). Measurements of chamber size were obtained from the endocardial surface of the ventricular septum to the endocardial surface of the ventricular wall (inner-edge to-inner edge).

Primary measurements recorded from the two-dimensional echocardiography (2-D) included aortic (AoD) and left atrial diameter (LAD) The frame from right parasternal short axis views optimized for aorta, just after aortic valve closure was selected with three aortic valve cusps seen with symmetry.

**Fig 3:**
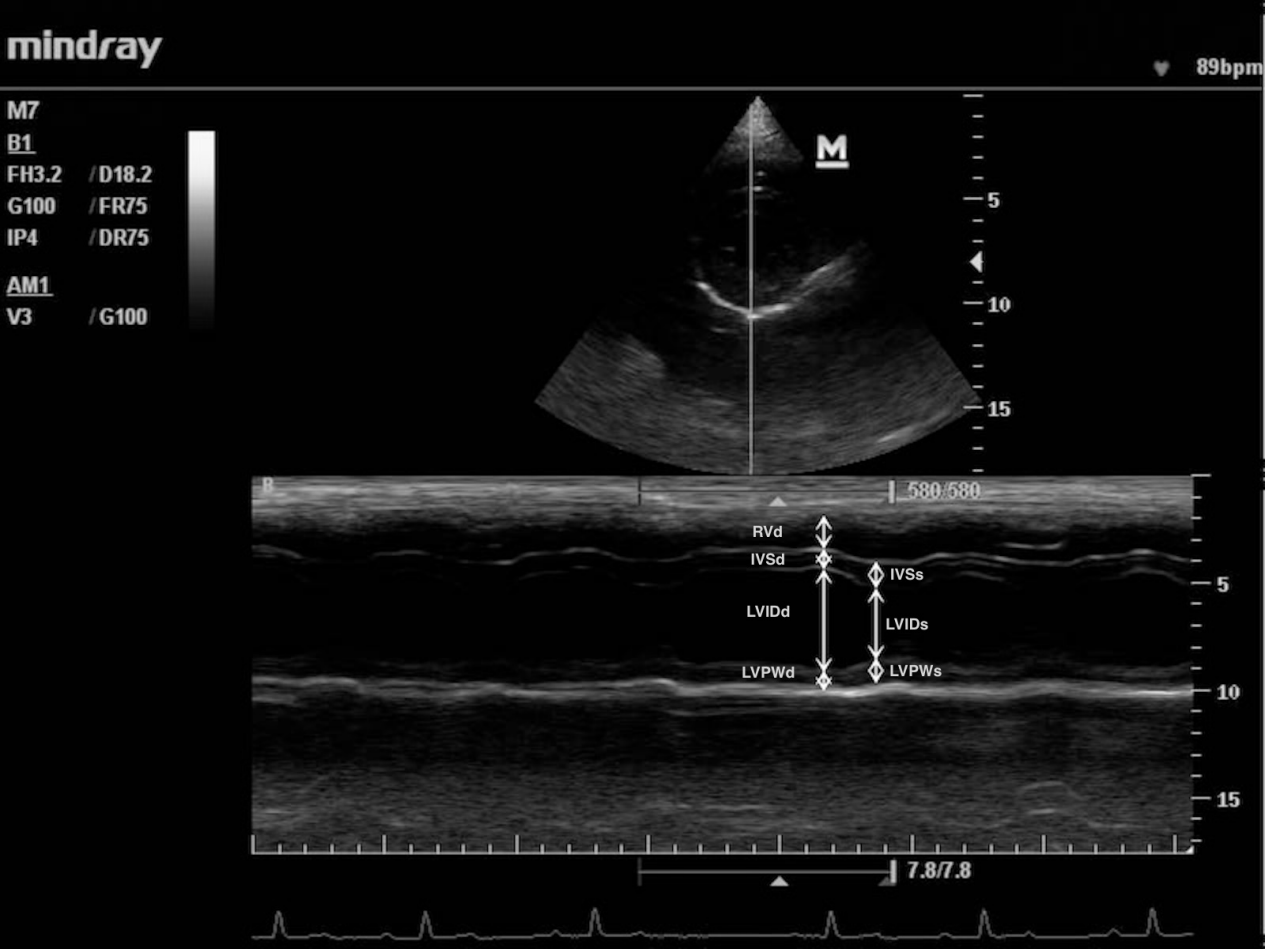
M-mode of the right and left ventricle obtained from the right parasternal short-axis view at the level of papillary muscles. RVd- right ventricle, IVSd- inter-ventricular septum, LVIDd—left ventricular internal diameter, LVPWd- left ventricular posterior wall thickness in diastole respectively, IVSs- inter-ventricular septum, LVIDs—left ventricular internal diameter, LVPWs- left ventricular posterior wall thickness in systole respectively.

Primary measurements recorded from the one-dimensional (M-mode) echocardiography included right ventricular internal diameter in diastole (RVIDd), left ventricular internal diameter in systole (LVIDs) and diastole (LVIDd), inter-ventricular septum thickness in systole (IVSs) and diastole (IVSd), left ventricular posterior wall thickness in systole (LVPWs) and diastole (LVPWd) and E-point to septal separation (EPSS)(Fig 3).

Primary measurements recorded from the Pulsed wave Doppler echocardiography included maximum Left and maximum Right Ventricular Outflow Tract velocity (LVOT Vmax and RVOT Vmax).

All measurements were taken in triplicate by the same person who performed echocardiography (OSJ) and then an averaged value was used in further calculations.

Derived values computed using the aforementioned primary measurements were:

1. the ratio of the left atrial diameter to the aortic diameter (LAD/AoD),
2. left ventricular fractional shortening (FS) calculated in M-mode with the integrated calculation program according to an equation: FS = 100%×(LVIDd-LVIDs)/LVIDd,
3. left ventricular ejection fraction (LVEF) calculated in M-mode with the integrated calculation program according to an equation: LVEF = [(7×LVIDd^3^)/(2.4+LVIDd)-(7×LVIDs^3^)/(2.4+ LVIDs)]×[100/(7×LVIDd^3^)/(2.4+LVIDd)].
4. maximum Left Ventricular Outflow Tract pressure gradient (LVOT PGmax): LVOT PGmax = LVOT Vmax^2^×4 and
5. maximum Right Ventricular Outflow Tract pressure gradient (RVOT PGmax): RVOT PGmax = RVOT Vmax^2^×4.

Animals with valvulopathy, hydropericardium or hydrothorax, heart wall deformation, any inclusions in the heart cavities (whose origin was not a subject of any further investigation) and turbulent blood flow inside the heart visible in the echocardiography were excluded from the study.

Guidelines for obtaining echocardiograms based on external anatomical landmarks were adapted from the ones previously described [17]. Time required for image acquisition was recorded.

### Statistical analysis

Numerical variables were given as an arithmetic mean and standard deviation (SD) if normally distributed, otherwise as a median and interquartile range). Normality of variable distribution was assessed using a Shapiro-Wilk W test. The range of values was always reported. Age and body weight were compared between the two goat breeds as well as between goats selected for repeatability assessment and the remaining goats using a Mann-Whitney U test. Analysis of covariance (ANCOVA) was used for comparison of echocardiographic measurements between the two breeds with control for the role of body weight. A p-value below 0.05 was considered to indicate statistical significance. Statistical analysis was performed in Statistica 10 (StatSoft Inc.).

Each measurement was investigated for extreme observations (outliers) using Tukey's rule and an observation was identified as extreme if its value was <Q1–3×IQR or >Q3 + 3×IQR. If a goat lacked more than one primary measurement it was excluded from the analysis. Unless missing data constituted more than 10% of all observations for a given measurement they were replaced with a respective arithmetic mean. Reference intervals (RIs) were calculated in the Reference Value Advisor [18] with the nonparametric method and presented as the lower and upper reference limit along with their 90% confidence intervals (90% CI) calculated using bootstrap method for EPSS [12] or nonparametric method in all the remaining cases. For these measurements which turned out to differ significantly between the two goat breeds when adjusted by the body weight, RIs were computed separately for each breed.

To assess the repeatability of the echocardiographic examination performed by the examiner involved in this study (OSJ) two approaches were applied. First, between-day coefficient of variation (CV) was calculated by dividing the overall standard deviation of the two repetitions by their mean [19] with its CI computed according to Zar[20]. Variability was classified as very low (<5%), low (5–15%), moderate (15–25%) and high (>25%) following classes proposed by Leroux et al. [4]. The upper limit of the 95% CI for CV determined the class to which CV belonged.

Secondly, the coefficient of repeatability (CoR) was determined. CoR was calculated from the following equation: within-subject SD×√2×1.96 [21]. Within-subject SD was estimated by fitting a one-way analysis of variance (ANOVA) model to the data containing the repeated measurements made on each subject and it was a square root of the mean within(i.e. error)-sum of squares. 95% CIs were calculated according to Bartlett and Frost [21]. CoR was given in the same units as the relevant measurement and it was defined as the least actual difference which could be detected using echocardiography performed by the examiner involved in this study (i.e. the observed value of the measurement is at least 95% likely to be different from the actual value of the measurement by no more than the value of CoR). To illustrate the actual magnitude of CoRs a percentage of the mean value of the measurement which a given CoR constitutes was also presented.

## Results

### Study population

Total number of 150 goats were examined and 20 did not meet medical eligibility criteria: 10 had hydropericardium (less than 10mm wide in all cases), 4 had mitral valve regurgitation, 3 had a heart rate over 150 bpm, 2 had aortic valve regurgitation, and one had a mediastinal tumor.

In total 6 outliers were found (one in RVIDd, RVOT Vmax, and two in EPSS and RVOT PGmax) and they were removed from a database turning into missing data. As a result five goats were dropped due to missing data and 125 goats were eventually included in the analysis. Twenty two of them lacked one primary measurement– 1 LVOT Vmax (0.8% of all cases), 4 RVOT Vmax (3.2%) and 17 EPSS (13.6%).

One hundred twenty five goats were included in the final analysis. Sixty four (51%) belonged to PFI breed and sixty one (49%) to PWI. Goats’ age ranged from 2 to 9 years and was non-normally distributed (p<0.001) with the median of 4 years, IQR from 3 to 5 years and no difference between the breeds (p = 0.898). Neither did body weight have normal distribution (p<0.001). PWI goats weighed between 48–80 kg (median of 67.9) and were significantly heavier than PFI goats which weighed between 46 and 73 kg (median of 58.5) (p = 0.001).

Twenty goats selected for the repeatability assessment had neither different body weight (p = 0.985) nor age (p = 0.491) compared to the remaining 105 goats.

### Reference intervals

In 19 cases primary measurements could not be taken due to the low quality of views (1 LVOT Vmax, 3 RVOT Vmax and 15 EPSS). RIs for echocardiographic measurements were calculated on 125 goats except for EPSS for which RI was determined on 108 goats as more than 10% of goats lacked this measurement.

RIs with 90% CI were determined for all 17 echocardiographic measurements (Table 1).

**Table 1:** Reference intervals of 17 2D, M-mode and pulsed wave Doppler echocardiographic measurements in 125 healthy adult dairy goats.

Echocardiographic measurement	Units	Shapiro-Wilk W test p-value	Descriptive statistics		Reference Interval	
			Mean (SD) or median (IQR)	Range	Lower limit (CI 90%)	Upper limit (CI 90%)
LAD	cm	0.279	3.29 (0.41)	2.30–4.64	2.52 (2.30, 2.64)	4.30 (4.03, 4.64)
AoD	cm	0.052	2.71 (0.27)	1.98–3.64	2.23 (1.98, 2.29)	3.29 (3.11, 3.64)
LAD/AoD	1/1	0.019*	1.21 (1.13–1.30)	0.83–1.76	0.96 (0.83, 1.01)	1.52 (1.45, 1.76)
RVIDd	cm	0.166	1.04 (0.36)	0.27–1.82	0.36 (0.27, 0.40)	1.67 (1.67, 1.82)
LVIDd	cm	0.563	4.17 (0.48)	2.81–5.69	3.20 (2.81, 3.34)	5.16 (4.93, 5.69)
LVIDs	cm	0.869	2.48 (0.38)	1.37–3.66	1.77 (1.37, 1.84)	3.24 (3.10, 3.66)
LVPWd	cm	0.075	0.87 (0.17)	0.48–1.37	0.53 (0.48, 0.64)	1.21 (1.16, 1.37)
LVPWs	cm	0.336	1.15 (0.21)	0.68–1.75	0.76 (0.68, 0.83)	1.59 (1.52, 1.75)
IVSd	cm	0.087	0.84 (0.14)	0.53–1.21	0.53 (0.53, 0.61)	1.14 (1.14, 1.21)
IVSs	cm	0.002*	1.04 (0.96–1.14)	0.61–1.59	0.69 (0.61, 0.80)	1.52 (1.37, 1.59)
EPSS	cm	0.011*	0.53 (0.46–0.61)	0.30–0.88	0.34 (0.30, 0.38)	0.83 (0.77, 0.88)
LVEF	%	0.726	71.1 (7.4)	50.8–89.7	55.5 (50.8, 59.6)	85.9 (83.2, 89.7)
FS	%	0.616	40.5 (6.3)	25.9–60.3	28.0 (25.9, 31.6)	53.8 (51.8, 60.3)
LVOT Vmax	cm/s	0.80	109 (16)	73–153	79 (73, 84)	141 (136, 153)
LVOT PGmax	mmHg	0.027*	4.74 (3.82–5.66)	2.15–9.33	2.52 (2.15, 2.82)	7.93 (7.35, 9.33)
RVOT Vmax	cm/s	0.759	97 (15)	62–138	69 (62, 73)	127 (121, 138)
RVOT PGmax	mmHg	0.019*	3.75 (3.00–4.58)	1.53, 7.62	1.94 (1.53, 2.12)	6.51 (5.84, 7.62)

* measurements non-normally distributed according to a Shapiro-Wilk W test and therefore described using a median and interquartile range (IQR)

Time required for image acquisition ranged from 11 to 39 min. with the median of 19 min.

### Repeatability

CVs for echocardiographic measurements ranged from 3.7% to 26%, however they mainly belonged to the class indicating low variability (10 of 17 CVs), followed by moderate variability (6 of 17). Only CV for EPSS indicated high variability (Table 2). Mean differences between the repeated examinations were equal to zero for all measurements which allowed calculation of relevant CoRs. CoRs along with their 95% CIs are presented in Table 3.

**Table 2:** Between-day coefficient of variation (CV) and 95% confidence intervals (95% CI) of echocardiographic measurements performed in 20 adult goats on two consecutive days.

Echocardiographic measurement	Units	Mean (SD)	CV(%)	95% CI for CV	Variability^a^
LAD	cm	3.43 (0.19)	5.5%	3.7%, 7.2%	low
AoD	cm	2.73 (0.14)	5.2%	3.5%, 6.8%	low
LAD/AoD	1/1	1.26 (0.09)	7.4%	5.0%, 9.7%	low
RVIDd	cm	1.18 (0.17)	14.4%	9.8%, 19.1%	moderate
LVIDd	cm	4.24 (0.25)	5.9%	4.0%, 7.7%	low
LVIDs	cm	2.59 (0.18)	7.0%	4.8%, 9.3%	low
LVPWd	cm	0.89 (0.13)	15.0%	10.1%, 19.8%	moderate
LVPWs	cm	1.19 (0.15)	12.5%	8.5%, 16.5%	moderate
IVSd	cm	0.9 (0.11)	12.7%	8.6%, 16.8%	moderate
IVSs	cm	1.12 (0.12)	10.4%	7.0%, 13.7%	low
EPSS	cm	0.54 (0.11)	19.8%	13.3%, 26.4%	high
LVEF	%	69.16 (5.08)	7.3%	5.0%, 9.7%	low
FS	%	39 (3.88)	9.9%	6.8%, 13.1%	low
LVOT Vmax	cm/s	107 (9)	8.6%	5.8%, 11.3%	low
LVOT PGmax	mmHg	4.62 (0.81)	17.6%	11.8%, 23.3%	moderate
RVOT Vmax	cm/s	97 (9)	9.5%	6.5%, 12.5%	low
RVOT PGmax	mmHg	3.83 (0.71)	18.5%	12.4%, 24.6%	moderate

^a^ classes of variability according to Leroux et al. [4]: <5% very low, 5–15% low, 15–25% moderate, >25% high–the upper limit of the 95% confidence interval for CV determines the class to which it belongs

**Table 3:** Coefficient of repeatability (CoR) with 95% confidence intervals (95% CI) of echocardiographic measurements performed in 20 adult goats on two consecutive days; mean difference between the two consecutive echocardiographic examinations with standard deviation (SD) and 95% confidence intervals (95% CI), and result of one sample Student’s t-test are also presented (α = 0.05).

Echocardiographic measurement	Units	Mean (SD) difference	95% CI for the mean difference	p-value of one-sample t-test	CoR	95% CI for CoR
LAD	cm	-0.08 (0.26)	-0.21, 0.04	0.157	0.52	0.40, 0.75
AoD	cm	0.00 (0.21)	-0.10, 0.09	0.976	0.39	0.30, 0.57
LAD/AoD	1/1	-0.03 (0.13)	-0.09, 0.03	0.345	0.26	0.20, 0.37
RVIDd	cm	-0.05 (0.24)	-0.16, 0.06	0.381	0.47	0.36, 0.68
LVIDd	cm	-0.07 (0.35)	-0.23, 0.10	0.408	0.69	0.53, 1.00
LVIDs	cm	-0.01 (0.26)	-0.13, 0.12	0.927	0.51	0.39, 0.73
LVPWd	cm	-0.06 (0.18)	-0.14, 0.03	0.179	0.37	0.28, 0.53
LVPWs	cm	0.00 (0.22)	-0.10, 0.10	0.967	0.41	0.32, 0.60
IVSd	cm	-0.04 (0.16)	-0.12, 0.03	0.240	0.31	0.24, 0.45
IVSs	cm	-0.03 (0.17)	-0.11, 0.05	0.392	0.32	0.25, 0.47
EPSS	cm	-0.05 (0.15)	-0.12, 0.02	0.130	0.30	0.23, 0.43
LVEF	%	-1.32 (7.24)	-4.71, 2.07	0.425	14.07	10.77, 20.32
FS	%	-1.19 (5.50)	-3.76, 1.39	0.347	10.76	8.23, 15.53
LVOT Vmax	cm/s	4 (13)	-2, 10	0.188	25	19, 37
LVOT PGmax	mmHg	0.33 (1.13)	-0.20, 0.86	0.205	2.25	1.72, 3.25
RVOT Vmax	cm/s	4 (13)	-2, 10	1,41	26	20, 37
RVOT PGmax	mmHg	0.33 (0.97)	-0.13, 0.78	0.149	1.96	1.50, 2.84

### Differences between the two breeds

Most of differences observed between PWI and PFI goats proved to result from the higher body weight of the former breed (Table 4). Only right ventricle was on average significantly wider in PWI goats (p = 0.018; RI from 0.33 to 1.78cm vs. 0.33 to 1.67cm in PFI), more than could be linked to their higher body weight. On the other hand PFI goats had slightly higher average systolic function indices (LVEF and FS), when adjusted by body weight. However, this difference disappeared when RIs were computed separately for the two breeds: LVEF: RI from 54.2% to 86.1% in PFI vs. 55.2% to 86.9% in PWI (p = 0.021), and FS: RI from 27.8% to 54.5% in PFI vs. 28.2% to 56.8% in PWI (p = 0.022).

**Table 4:** Comparison of echocardiographic measurements between Polish White Improved (PWI) and Polish Fawn Improved (PFI) goats.

Echocardiographic measurement	Units	Breed (mean±SD)		ANCOVA (p-value)	
		PWI (n = 61)	PFI (n = 64)	Breed	Body weight
LAD	cm	3.36±0.40	3.26±0.41	0.634	0.042*
AoD	cm	2.80±0.28	2.64±0.22	0.588	0.006*
LAD/AoD	1/1	1.21±0.13	1.24±0.14	0.389	0.962
RVIDd	cm	1.15±0.37	0.95±0.33	0.018*	0.838
LVIDd	cm	4.29±0.49	4.07±0.43	0.646	0.025*
LVIDs	cm	2.57±0.36	2.4±0.39	0.073	0.972
LVFWd	cm	0.91±0.19	0.84±0.14	0.885	0.016*
LVFWs	cm	1.21±0.21	1.11±0.18	0.675	0.019*
IVSd	cm	0.88±0.14	0.81±0.13	0.050	0.858
IVSs	cm	1.10±0.19	1.03±0.15	0.607	0.134
EPSS	cm	0.55±0.12	0.52±0.11	0.623	0.623
LVEF	%	70.48±7.04	71.81±7.68	0.021*	0.028*
FS	%	40.02±6.07	41.05±6.45	0.022*	0.023*
LVOT Vmax	cm/s	110±14	109±17	0.213	0.144
LVOT PGmax	mmHg	4.89±1.28	4.85±1.49	0.257	0.146
RVOT Vmax	cm/s	100±15	95±14	0.185	0.981
RVOT PGmax	mmHg	4.09±1.25	3.67±1.06	0.172	0.992

* statistically significant differences (p<0.05).

## Discussion

The study allowed to determine reference intervals for 17 main echocardiographic measurements. Given the large number of animals enrolled, and the use of both commonly accepted echocardiographic procedures and recommended statistical method, these RIs are very likely to show true normal echocardiographic measurements of clinically healthy goats. All the goats in this study were in lactation, however no differences between echocardiographic measurements taken during lactation, pregnancy or dry period have been so far revealed [2]. Therefore, RIs determined in the current study can be applied to all female adult dairy goats regardless of the production cycle stage.

Generally, they are in agreement with RIs for a mixed sample of two breeds (Saanen and Golden Guernsey) previously reported by Hallowell et al. [7], although they are usually wider, which is likely to result, at least partly, from a different method of RIs calculation (non-parametric in our study versus parametric in the previous one). Slight differences in heart wall and chamber dimensions can be attributed to the higher body weight of goats in the current study. The only noteworthy exception is LVEF whose lower reference limit is much lower in the current study (55% vs. 63%). Two measurements are considered as indicators of systolic function in dogs and humans–FS and LVEF. From among them RI for FS obtained in this study is comparable with RIs for dogs [22] and people [23]. On the other hand, LVEF’s lower limit is much closer to the lower limit commonly accepted in dogs and in humans as a critical value indicating abnormally weak heart function and heart failure [22–24].

Variability of measurements in the current study was comparable with results of Leroux et al. [4,5] when expressed as CV. However, this study is the first in which repeatability of echocardiographic examination was also assessed according to commonly acknowledged statistical rules [21, 25] i.e. using the coefficient of repeatability. This measure has more intuitive medical interpretation than CV, since it is simply the prediction interval in which the actual value of the measurement is 95% certain to lie given a particular observed value of this measurements. Undeniably, CoRs provide considerably less optimistic view of precision assured by the echocardiography than other measures of variability previously presented such as intra-class correlation coefficient [7] or coefficient of variation [4, 5]. CoRs are much wider e.g. FS whose RI is 28–54% is actually given with ±11% precision. Obviously repeatability strongly depends on the echocardiographer’s professional expertise and manual skills and such huge imprecision may easily be attributed to the lack these virtues. However, the examiner involved in the current study had intensively practiced as an echocadiographer for 10 years, including 2-year practice in goats, before the current study commenced. Hence, blaming the examiner for high CoRs would be unjustified. More likely is the conception that this is how the reality of a daily echocardiography practice appears when limited cooperation of a patient can be expected. In the current study we tried to reproduce natural conditions in which the echocardiography is performed in animals routinely examined cardiologically such as dogs or cats–examinations were performed carefully, in barns were animals regularly live and in the presence of their everyday caretakers but in a routine manner with attention paid not only to precision but also to efficiency of the whole procedure. Goats differed with respect to their cooperation (despite our efforts to reduce their stress some showed marked excitement and had to be held tightly by their caretakers) as well as their body conformation (body score from 2 to 4), and these factors could have affected the precision of measurements obtained and resulted in quite wide RIs. Moreover, we had to spend quite long time on examining each goat (three goats nearly 40 minutes to acquire acceptable images). However, we believe that selection of only the most cooperative goats would bias the results, especially would it falsely inflate the repeatability coefficients.

Obviously, the technique of echocardiographic examination strongly affected the RIs and CoRs provided in our study and they are likely to differ when a different technique is used. To allow for their broadest use, we did our utmost to perform echocardiography according to widely accepted standards.

Goats of two local Polish breeds were enrolled in the study, which may limit applicability of the results. However, both breeds were developed in last two decades of the 20^th^ century by mating native goats with two popular dairy breeds–French Alpine (for PFI) and Saanen (for PWI) [26]. Actually, many goats included in the study were closely related to either French Alpine or Saanen goats as they came from highly-producing dairy farms, where regular breeding policy aimed to maximize milk productivity by mating with the aforementioned pedigree goats. The share of genes of improving breeds was even up to 80% in the herd in which more than 50% of goats used in this study were kept [27]. The analysis showed that differences between the two breeds could be attributed to different body weights with only three exceptions (RVIDd, LVEF and FS) in which differences although statistically significant were unlikely to be clinically relevant since the RIs computed separately for the two breeds mostly overlapped.

### Conclusion

The study provides echocardiographic RIs for goats determined using the highest so far enrolled group of clinically healthy animals.
